# Routinely available inflammation biomarkers to predict stroke and mortality in atrial fibrillation

**DOI:** 10.1016/j.clinsp.2025.100610

**Published:** 2025-03-11

**Authors:** Long Wu, Zhiquan Yuan, Yuhong Zeng, Lanqing Yang, Qin Hu, Huan Zhang, Chengying Li, Yanxiu Chen, Zhihui Zhang, Li Zhong, Yafei Li, Na Wu

**Affiliations:** a Department of Epidemiology, College of Preventive Medicine, Army Medical University (Third Military Medical University), PR China; b Evidence-based Medicine and Clinical Epidemiology Center, Army Medical University (Third Military Medical University), PR China; c Department of Cardiology and the Center for Circadian Metabolism and Cardiovascular Disease, Southwest Hospital, Army Medical University (Third Military Medical University), PR China; d Department of Cardiology, Third Affiliated Hospital of Chongqing Medical University, PR China

**Keywords:** Atrial fibrillation, Inflammation biomarker, Stroke, Mortality, CHA_2_DS_2_-VASc

## Abstract

**Background:**

This study aimed to assess the predictive value of 7 routinely available inflammation biomarkers for stroke and all-cause mortality in 229 non-valvular AF patients.

**Methods and results:**

C-reactive protein, Albumin (ALB), d-dimer, fibrinogen, the number of platelets, lymphocytes, monocyte and neutrophils were measured. The Multivariable Cox proportional hazard model was used to assess the predictive value of the inflammation biomarkers for stroke and all-cause mortality, the c-statistic, Net Reclassification Improvement (NRI), and Integrated Discrimination Improvement (IDI) were calculated. Lymphocyte Monocyte Ratio (LMR) was the most informative biomarker for predicting stroke, and adding LMR to the CHA_2_DS_2_-VASc score did not improve its predictive ability for stroke, but it did improve its reclassification ability. Similar results were observed when comparing LMR+ CHA_2_DS_2_-VASc score with the ABC stroke score. For all-cause mortality, a Systemic Inflammation Score (SIS) score was calculated using ALB, fibrinogen and LMR, and adding SIS to the CHA_2_DS_2_-VASc score showed a significant improvement in its predictive ability and reclassification ability. There were no significant differences in predictive and reclassification ability for all-cause mortality between SIS+CHA_2_DS_2_-VASc score and ABC death score.

**Conclusions:**

Adding routinely available inflammatory biomarkers to the CHA_2_DS_2_-VASc score increased its ability to predict all-cause mortality, suggesting this cost-effective biomarker strategy may help to improve decision support in AF.

## Highlights

• Adding Lymphocyte to Monocyte Ratio (LMR) to CHA_2_DS_2_-VASc score improves its reclassification ability and is comparable with the ABC score in predicting stroke.
• Adding a systemic inflammation score (using LMR, albumin and fibrinogen) to CHA_2_DS_2_-VASc score improves its predictive and reclassification ability and is comparable with the ABC score in predicting death.
• Because traditional inflammatory biomarkers are more readily available and less expensive, they may be used to improve decision support for AF.

## Introduction

Atrial Fibrillation (AF) is one of the most common clinical arrhythmias^1^^,^^2^ and is associated with increased risks of ischemic stroke and all-cause mortality. AF may result in impaired quality of life and higher medical care costs.^3^

To prevent stroke and improve the survival of AF patients, it is important to accurately predict adverse outcomes at an early stage before severe damage has occurred.^4^ Risk stratification schemes for AF patients have been proposed since the 1990s.^5^ The CHA_2_DS_2_-VASc score is the most widely used stratification scheme for stroke risk and is recommended by the current guidelines.^6^^,^^7^ However, the CHA_2_DS_2_-VASc score has only modest predictive value for identifying subjects with a high risk of stroke and who therefore need an oral anticoagulant, with a c-statistic of approximately 0.60.^8^ A number of risk prediction tools were recently developed for estimating the mortality risk in AF,^9^, ^10^, ^11^ but there is still no commonly accepted tool. Several studies have evaluated the CHA_2_DS_2_-VASc score for predicting mortality risk.^12^, ^13^, ^14^

Over the past few decades, various biomarkers have been identified that might have value for predicting AF and related outcomes.^15^ Some studies demonstrated that the addition of several biomarkers, such as NT-proBNP, hs-troponin T, d-dimer, IL-6 and troponin I, could improve the accuracy of the CHA_2_DS_2_-VASc score for predicting stroke and mortality in AF patients.^12^^,^^13^^,^^16^, ^17^, ^18^, ^19^ Hijazi et al. recently developed the ABC risk score, which includes age, several biomarkers (NT-proBNP, Troponin T / Troponin I, growth differentiation factor-15), and clinical history. Internal and external validation studies have reported the ABC-stroke and ABC-death scores to show higher c-indices than the CHA_2_DS_2_-VASc score.^9^^,^^20^

Cost-effectiveness must be considered for the incorporation of biomarkers as risk prediction tools.^15^ Inflammation has a possible pathogenic link to AF prognosis,^21^ and a routinely available inflammation biomarker is one of the most important biomarkers available. Complete Blood Count (CBC) is a common and inexpensive diagnostic method used in routine practice. Neutrophils, lymphocytes, monocytes and platelets play a central role in inflammation.^22^ The Platelet to Lymphocyte Ratio (PLR), Lymphocyte to Monocyte Ratio (LMR) and Neutrophil to Lymphocyte Ratio (NLR) can be calculated from the CBC and have been used as indicators of systemic inflammation in numerous studies.^23^^,^^24^ C-Reactive Protein (CRP), Albumin (ALB), fibrinogen, and d-dimer are also closely related to inflammation and have been associated with stroke and mortality in AF patients.^21^^,^^25^, ^26^, ^27^, ^28^, ^29^, ^30^, ^31^, ^32^, ^33^, ^34^

The 7 biomarkers described above (PLR, LMR, NLR, CRP, ALB, fibrinogen, and d-dimer) are all routinely available markers of inflammation. They are less expensive and more readily available in routine clinical practice than the biomarkers included in the ABC score. The authors, therefore, conducted this study to identify routine inflammation biomarkers with the highest predictive ability for stroke and all-cause mortality in AF. The authors also tested the hypotheses that the addition of routinely available inflammation biomarker(s) to the CHA_2_DS_2_-VASc score could improve its ability to predict stroke or all-cause mortality and that the addition of inflammation biomarkers to CHA_2_DS_2_-VASc score would be comparable with the ABC score in predicting stroke or all-cause mortality.

## Methods

### Study population

Study participants were consecutively recruited from the inpatients of a large hospital from December 2016 to January 2018. Inclusion criteria were newly diagnosed AF patients aged at least 18-years, and with complete medical records. Exclusion criteria included the occurrence of a cerebrovascular event during hospitalization, the presence of heart valve disorder, infectious disease, chronic inflammatory disease, acute inflammatory disease, malignancy, or connective tissue disease. AF was diagnosed according to the 2010 ESC guidelines. Structured interviews and medical records were used to collect demographic and clinical information. Drink status was classified as a current drinker, past drinker and non-drinker. A current drinker was defined as drinking alcohol at least once a week and continued to drink during the past 30-days. Past drinkers were those who drank alcohol at least once in their lives but did not consume alcohol in the past 30-days. Non-drinkers were defined as participants who had never consumed alcohol. In the analysis, drink status was classified as non-drinker and drinker (including current and past drinkers).^35^ The study was approved by the Ethics Committee of the Southwest Hospital of Army Medical University (KY2020231), and all participants provided informed consent.

### Study endpoints and follow-up

The two primary endpoints were ischemic stroke and all-cause mortality. Ischemic stroke was defined as having one inpatient, emergency room, or outpatient claim with primary or secondary ICD10 code I63. All participants were followed up once a year by telephone. Information on endpoints was confirmed by screening the medical records and the national register of death.

### Measurement of inflammation biomarkers

Circulating levels of inflammation biomarkers were measured. A total of 5 mL of fasting blood was drawn from each patient within 48 h of admission before any treatment. CRP and fibrinogen were measured by IMMAGE 800 specific protein analyzer (Beckman Coulter, USA). ALB, d-dimer, platelets, lymphocytes, monocytes and neutrophils were measured by XS800i Hematology Analyzer (Sysmex, Japan). PLR, LMR and NLR were calculated using the numbers of platelets, lymphocytes, monocytes and neutrophils. The biomarkers used for the ABC score were NT-proBNP, cTnT, cTnI and GDF-15. NT-proBNP, cTnT and cTnI were measured by Electro-Chemiluminescence Immunoassay (ECLIA) on Cobas e601 (Cobas e601, Rocha Diagnostics, Manheim, Germany) with a lower limit of detection of 5.0 pg/mL and 3.0 ng/L. GDF-15 was measured by ELISA kit (Raybiotech, Norcross, GA, USA). The detection range was 2‒800 pg/mL, and the inter- and intra-assay coefficients of variation were <10.0 % and <12.0 %, respectively.

### Statistical analysis

The descriptive statistics are presented as frequency counts and proportions for categorical data, as the mean and Standard Deviation (SD) for continuous variables with a normal distribution, and as the median and interquartile range (25th‒75th percentile) for continuous variables with a non-normal distribution.

Circulating inflammation biomarkers’ values were transformed into dichotomous variables based on the cut-off values using the X-tile program 3.6.1 (Yale University).^36^ The optimal cut-off point was determined according to the composite outcome (stroke or all-cause mortality). Detailed cut-off values for each biomarker are shown in Supplementary Table 1. Subsequently, univariable Cox modeling was used to assess crude associations between each baseline variable including the 7 inflammation biomarkers, and stroke/all-cause mortality. A value of *p* < 0.1 was set in the univariable Cox model to select variables for inclusion in the multivariable Cox model.^37^, ^38^, ^39^ Backward elimination (p for retention in model = 0.05) was then performed to identify the most important variables in the multivariable Cox model.^40^^,^^41^ The inflammation biomarkers retained in the final model were those with the highest predictive ability for stroke and all-cause mortality in AF. Sensitivity analyses included the inclusion of warfarin and statins to the model Cox model or using forward selection. Finally, a Systemic Inflammation Score (SIS) was developed to assess the combined effect of the selected inflammation biomarkers that included in the final model. The weighted points were estimated using the absolute value. SIS=(|λ1| × biomarker-*A*+(|λ2| × biomarker-*B*+(|λ3| × biomarker-C), and so on, where λ1, λ2, and λ3 denote the weighted points for biomarkers-A, -B, and -C. λ was calculated according to estimates of the beta coefficient (β) for biomarkers by fitting the final Cox model. To simplify the weighted points, the other β was divided by the β whose absolute value was the smallest, and rounded the quotients up to the nearest integer. The λ was set as 1 for the biomarker whose absolute value of β was the smallest. The detailed method is shown in Supplementary Table 2 and Supplementary Table 3. Kaplan-Meier estimates of stroke and all-cause mortality according to SIS were calculated and plotted.

To test the hypothesis that addition of the routinely available inflammation biomarkers to the CHA_2_DS_2_-VASc score can improve its predictive ability, the authors assessed the improvement in discrimination and reclassification, which are two elements of predictive ability. For the discrimination, the c-statistic for the predictive models was calculated. The c-statistic of the two models was compared as described by Hanley and McNeil.^42^ Net Reclassification Improvement (NRI) and Integrated Discrimination Improvement (IDI) were calculated to assess if the inclusion of LMR or SIS improved risk reclassification.

A two-sided p-value < 0.05 was considered to be statistically significant. All statistical analyses were conducted using R 3.6.2 (R Foundation for Statistical Computing, Vienna, Austria).

## Results

### Clinical characteristics

In total, 232 newly diagnosed non-valvular AF patients were enrolled in the study, of whom 3 (1.3 %) were loss to follow-up. During the median follow-up of 26-months (IQR, 22‒29), 21 patients developed stroke (incidence rate of 4.64/100 person-years) and 26 patients died (incidence rate of 5.47/100 person-years).

The median age of patients was 67-years (IQR, 59‒75 years), and 54.6 % were male (Table 1). Chronic AF accounted for 69.9 % of all patients. The median CHA_2_DS_2_-VAS_C_ score was 2 (IQR, 1‒4), and 69.4 % of patients had a CHA_2_DS_2_-VAS_C_ score ≥2. The median one-year risk of stroke estimated by the ABC stroke score was 1.1 % (IQR, 0.8 %‒1.7 %), while the one-year risk of mortality estimated by the ABC death score using cTnT was 1.7 % (IQR, 0.9 %‒4.0 %).

**Table 1:** Demographics and baseline characteristics of AF patients.

Characteristics	Values (*n* = 229)
Age (years)	67 (59, 75)
Gender (male/female)	125/104 (54.6 %/45.4 %)
BMI (kg/m^2^)	23.9 (21.9, 26.2)
Education	
Junior middle school or below	186 (81.2 %)
High school or above	43 (18.8 %)
Income per head (10000 yuan/year)	
< 2.5	106 (46.3 %)
≥ 2.5	123 (53.7 %)
AF types	
Paroxysmal AF	69 (30.1 %)
Chronic AF	160 (69.9 %)
Smoke	71 (31.0 %)
Drink	71 (31.0 %)
History of comorbidities	
Hypertension	118 (51.5 %)
Diabetes	38 (16.6 %)
CAD	87 (38.0 %)
Cardiomyopathy	24 (10.5 %)
HF	79 (34.5 %)
Vascular disease	16 (7.0 %)
Previous stroke	27 (11.8 %)
Concomitant treatment	
Warfarin	69 (30.1 %)
Statins	101 (44.1 %)
Echocardiography Parameters	
LA diameter (mm)	44 (40, 51)
LV diameter (mm)	49 (45, 54)
RA diameter (mm)	43 (35, 48)
RV diameter (mm)	20 (19, 23)
LVEF (%)	59 (49, 65)
Inflammation cytokines	
CRP (mg/L)	45.5 (24.8,70.9)
≤ 12.0	194 (89.4 %)
> 12.0	23 (10.6 %)
ALB (g/L)	7.9 (2.5,17.9)
≤ 38.2	121 (55.8 %)
> 38.2	96 (44.2 %)
Fibrinogen (g/L)	35.7 (13.0, 60.0)
≤ 3.3	190 (87.6 %)
> 3.3	27 (12.4 %)
D-dimer (mg/L)	0.3 (0.2,0.7)
≤ 0.6	165 (72.1 %)
> 0.6	64 (27.9 %)
PLR	102.8 (77.6,132.0)
≤ 91.2	92 (40.2 %)
> 91.2	137 (59.8 %)
LMR	3.8 (2.7,5.1)
≤ 2.7	55 (24.0 %)
> 2.7	174 (76.0 %)
NLR	2.2 (1.6, 3.4)
≤ 4.1	192 (83.8 %)
> 4.1	37 (16.2 %)
SIS for mortality	
0	96 (41.9 %)
1	88 (38.4 %)
2	34 (14.9 %)
3	11 (4.8 %)
CHA_2_DS_2_-VASc	2 (1,4)
CHA_2_DS_2_-VASc≥2	159 (69.4 %)
Biomarkers of ABC score proBNP (pg/mL)	1103 (522, 1999)
cTnT (ng/L)	0.01 (0.01, 0.02)
cTnI (ng/L)	0.01 (0.01, 0.02)
GDF-15 (pg/mL)	1018 (743, 1453)
One-year risk of stroke calculated by ABC stroke score	1.1 % (0.8 %, 1.7 %)
One-year risk of mortality calculated by ABC death score using cTnT	1.7 % (0.9 %, 4.0 %)
One-year risk of mortality calculated by ABC death score using cTnI	1.6 % (0.8 %, 3.6 %)
Follow-up time (months)	26 (22, 29)
Person-years of follow-up	
Stroke	452.7
All-cause mortality	475.4
Events (per 100 person-years)	
Stroke	4.6
All-cause mortality	5.5

Continuous variables are expressed as median and Interquartile Range (IQR). Categorical variables are expressed as frequencies and percentages.

AF, Atrial Fibrillation; BMI, Body Mass Index; CAD, Coronary Heart Disease; HF, Heart Failure; LA, Left Atrium; LV, Left Ventricle; RA, Right Atrium; RV, Right Ventricle; LVEF, Left Ventricular Ejection Fraction; CRP, C-Reactive Protein; ALB, Albumin; PLR, Platelet to Lymphocyte Ratio; LMR, Lymphocyte to Monocyte Ratio; NLR, Neutrophil Tolymphocyte Ratio; cTnT, cardiac Troponin T; cTnI, cardiac Troponin I; GDF-15; Growth Differentiation Factor-15; SIS, Systemic Inflammation Score.

### LMR is the most important inflammation biomarker for stroke

**Fig. 1:**
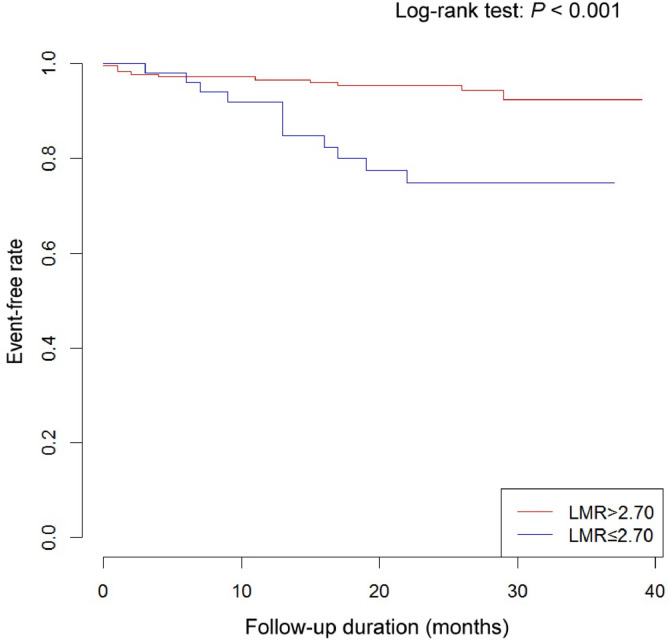
Kaplan-Meier plot of stroke event-free curves for different LMR levels. LMR, Lymphocyte to Monocyte Ratio.

Cox proportional hazard regression analysis was applied to assess the predictive value of 7 inflammation biomarkers for stroke and all-cause mortality in AF patients. The most important predictors of stroke were LMR, drink status, history of diabetes, history of previous stroke, and left atrium diameter, with a low level of LMR associated with an increased risk of stroke (Table 2). The results remained robust after the addition of warfarin and statin use to the model, or the adoption of forward selection (Supplementary Table 4). Kaplan-Meier analysis revealed a significant association (*p* < 0.001) between the LMR and the risk of stroke (Fig. 1).

**Table 2:** Association of inflammation biomarkers with adverse events.

	Multivariate Cox proportional hazard regression analysis^a^		
	Beta coefficients	Adjusted HR (95 % CI)	p-value
**Stroke**			
LMR			
≤ 2.70		Reference	
> 2.70	−1.68	0.19 (0.07, 0.47)	**<0.001**
**All-cause mortality**			
ALB			
≤ 38.2 g/L		Reference	
> 38.2 g/L	−1.14	0.32 (0.11, 0.95)	**0.041**
Fibrinogen			
≤ 3.3 g/L		Reference	
> 3.3 g/L	1.08	2.94 (1.23, 7.03)	**0.016**
LMR			
≤ 2.70		Reference	
> 2.70	−1.47	0.23 (0.09, 0.62)	**0.003**
SIS			
0		Reference	
1	1.34	3.83 (0.43, 34.07)	0.229
2	3.14	23.09 (2.94, 181.48)	**0.003**
3	4.05	57.63 (6.66, 499.20)	**<0.001**

HR, Hazard Ratio; ALB, Albumin; LMR, Lymphocyte to Monocyte Ratio.

The p-value with bold font indicate p-value < 0.05.

a Cox proportional hazards model for stroke adjusted for age, drink status, history of diabetes, history of previous stroke, right atrium diameter, left atrium diameter and these 7 biomarkers using a backward selection strategy, and drink status, history of diabetes, history of previous stroke, left atrium diameter and LMR were in the final model. For all-cause mortality, adjusted for age, history of heart failure, left atrium diameter, left ventricular diameter, right atrium diameter, right ventricular diameter and these 7 biomarkers using a backward selection strategy, and age, right ventricular diameter, ALB, fibrinogen and LMR were in the final model.

### ALB, fibrinogen and LMR are the most important inflammation biomarkers for all-cause mortality

**Fig. 2:**
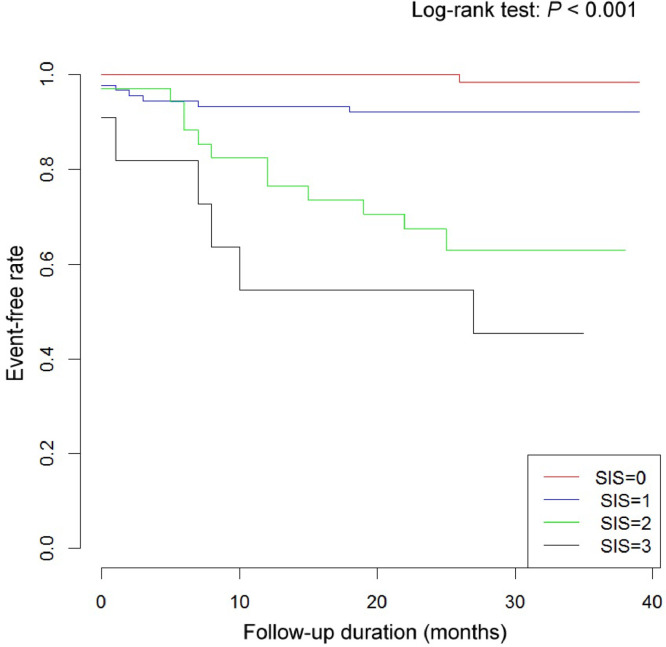
Kaplan-Meier plot of all-cause mortality event-free curves for systemic inflammation score. SIS, Systemic Inflammation Score.

ALB, fibrinogen, LMR, age and right ventricular diameter were identified as significant predictors of all-cause mortality in the final model. A high level of fibrinogen, low level of ALB, and low LMR were associated with an increased risk of mortality (Table 2), and sensitivity analyses showed the same results (Supplementary Table 4). The SIS for all-cause mortality was computed based on ALB, fibrinogen and LMR. Kaplan-Meier analysis confirmed the high discriminative power (*p* < 0.001) when plotting SIS with survival rates for all-cause mortality (Fig. 2).

### Adding LMR to CHA_2_DS_2_-VASc score did not improve its predictive ability but improved its reclassification ability for stroke

The authors next tested the hypothesis that the addition of LMR to CHA_2_DS_2_-VASc score would improve its predictive ability for stroke. The c-statistic for the prediction of stroke by the CHA_2_DS_2_-VASc score was 0.75 (95 % Confidence Interval [95 % CI: 0.67 to 0.82]). This increased to 0.79 (0.72 to 0.87) following the addition of LMR, but the improvement was not statistically significant 0.04 (−0.04 to 0.13; *p* = 0.286). However, the addition of LMR did lead to a significant improvement in reclassification, based on continuous NRI analyses 46.3 % (0.0 % to 70.5 %; *p* = 0.030), and the IDI 9.9 % (1.8 % to 24.5 %; *p* < 0.001) (Table 3).

**Table 3:** Predictive accuracy of different risk scores to predict adverse events.

	Discrimination				Reclassification			
	C-statistic (95 % CI)	p-value	Improvement in c-statistic (95 % CI)	p-value	NRI (95 % CI)	p-value	IDI (95 % CI)	p-value
**Stroke**								
CHA_2_DS_2_-VASc	0.75 (0.67, 0.82)	**<0.001**	‒	‒	‒	‒	‒	‒
ABC stroke risk score	0.78 (0.70, 0.86)	**<0.001**	‒	‒	‒	‒	‒	‒
CHA_2_DS_2_-VASc +LMR	0.79 (0.72, 0.87)	**<0.001**	0.04^a^ (−0.04, 0.13)	0.286	46.3 %^a^ (0.0 %, 70.5 %)	**0.030**	9.9 %^a^ (1.8 %, 24.5 %)	**<0.001**
			0.01^b^ (−0.16, 0.13)	0.866	46.3 %^b^ (20.2 %, 67.7 %)	**<0.001**	11.2 %^b^ (−3.0 %, 31.5 %)	0.109
**All-cause mortality**								
CHA_2_DS_2_-VASc	0.71 (0.64, 0.79)	**<0.001**	‒	‒	‒	‒	‒	‒
ABC death risk score using cTnT	0.84 (0.76, 0.91)	**<0.001**	‒	‒	‒	‒	‒	‒
ABC death risk score using cTnI	0.82 (0.74, 0.90)	**<0.001**	‒	‒	‒	‒	‒	‒
CHA_2_DS_2_-VASc +SIS	0.86 (0.81, 0.91)	**<0.001**	0.15^a^ (0.05, 0.25)	**0.003**	69.3 %^a^ (−27.3 %, 83.0 %)	0.090	25.1 %^a^ (7.4 %, 43.8 %)	**<0.001**
			0.03^c^ (−0.04, 0.09)	0.404	8.9 %^c^ (−41.0 %, 79.7 %)	0.687	9.8^c^ (−6.5 %, 27.8 %)	0.249
			0.04^d^ (−0.03, 0.12)	0.259	−34.8 %^d^ (−57.5 %, 75.9 %)	0.746	2.2 %^d^ (−13.8 %, 14.8 %)	0.726

LMR, Lymphocyte to Monocyte Ratio; cTnT, cardiac Troponin T; cTnI, cardiac Troponin I; NRI, Net Reclassification Improvement; IDI, Integrated Discrimination Improvement. The p-value with bold font indicate p-value < 0.05.

a Compared with CHA_2_DS_2_-VASc.

b Compared with ABC stroke risk score.

c Compared with ABC death risk score using cTnT.

d Compared with ABC death risk score using cTnI.

### Adding SIS to the CHA_2_DS_2_-VASc score improved its predictive ability for all-cause mortality

The c-statistic for all-cause mortality improved from 0.71 (0.64 to 0.79) for CHA_2_DS_2_-VASc score alone, to 0.86 (0.81 to 0.91) after the addition of SIS. This increase was statistically significant (0.15 [0.05 to 0.25], *p* = 0.003). The IDI also showed a significant improvement in reclassification with the addition of SIS (25.1 % [7.4 % to 43.8 %]; *p* < 0.001), while NRI showed a non-statistically significant increase (69.3 % [−27.3 % to 83.0 %]; *p* = 0.090) (Table 3).

### Adding LMR to the CHA_2_DS_2_-VASc score was comparable to the ABC stroke score in predicting stroke

Next, the authors evaluated whether there was a significant difference in the predictive ability for stroke between the ABC score and the CHA_2_DS_2_-VASc score + LMR. The c-statistic of the ABC score for the prediction of stroke was 0.78 (0.70 to 0.86), compared to 0.79 (0.72 to 0.87) after adding LMR to the CHA_2_DS_2_-VASc score. This increase was not statistically significant (0.01 [−0.16 to 0.13]; *p* = 0.866). However, the addition of LMR to the CHA_2_DS_2_-VASc score improved reclassification compared with the ABC stroke score, with an NRI of 46.3 % (20.2 % to 67.7 %; *p* < 0.001) and IDI of 11.2 % (−3.0 % to 31.5 %; *p* = 0.109) (Table 3).

### Adding SIS to the CHA_2_DS_2_-VASc score was comparable to the ABC death score in predicting all-cause mortality

The c-statistic for prediction of all-cause mortality by the ABC death score was 0.84 (0.76 to 0.91) with cTnT, and 0.82 (0.74 to 0.90) with cTnI. The addition of SIS to the CHA_2_DS_2_-VASc score (CHA_2_DS_2_-VASc+SIS) resulted in a c-statistic for all-cause mortality of 0.86 (0.81 to 0.91). No significant difference in the c-statistic was observed between the CHA_2_DS_2_-VASc+SIS score and the ABC death score. Furthermore, no significant differences in reclassification for NRI and IDI were observed between the CHA_2_DS_2_-VASc+SIS score and the ABC death score (Table 3).

## Discussion

This study found that LMR was the most informative biomarker for predicting stroke, while the strongest predictors for all-cause mortality were ALB, fibrinogen and LMR. A low LMR was associated with an increased risk of stroke. Lymphopaenia may suggest the immune response is suppressed, while monocytosis reflects chronic systemic inflammation.^43^ Overwhelming systemic inflammation and immune suppression have been shown to increase the risks of stroke and mortality.^27^^,^^44^ Low LMR has been associated with an increased risk of mortality in coronary heart disease,^43^ peripheral arterial disease,^45^ and malignancy.^46^^,^^47^ The Albumin (ALB) level tends to decline in inflammatory conditions.^48^ Low ALB level is associated with higher risks of mortality and thrombus in AF patients, probably due to systemic inflammation or malnutrition.^29^, ^30^, ^31^ Fibrinogen is a major coagulation factor and is also an effecter and stimulator of inflammatory reactions.^49^ This biomarker is a predictor of ischemic stroke and mortality in AF.^32^^,^^33^ However, published results have been inconsistent, probably due to differences in study populations, the time and method of fibrinogen measurement, and the length of follow up.^17^^,^^33^

In the present study, the addition of LMR did not significantly improve the predictive ability of the CHA_2_DS_2_-VASc score for stroke. This finding indicates the CHA_2_DS_2_-VASc score is still an appropriate risk stratification scheme for stroke. It can be easily calculated and provides a simple, quick and reliable initial assessment of risk in the acute stroke setting. The addition of SIS to the CHA_2_DS_2_-VASc score significantly improved its predictive value for all-cause mortality compared to the CHA_2_DS_2_-VASc score alone. No significant difference in predictive ability was observed between the CHA_2_DS_2_-VASc+SIS score and the ABC death score. However, the predictive ability improved from modest to good using CHA_2_DS_2_-VASc+SIS. The included biomarkers are more readily accessible than those in the ABC death score, indicating that CHA_2_DS_2_-VASc+SIS may allow cost-effective stratification for the risk of mortality in clinical practice.

The CHA_2_DS_2_-VASc score was originally developed to refine the assessment of stroke risk in patients with non-valvular AF. Although the guidelines began to recommend the use of this score in 2014,^7^ it has only modest predictive value. The authors of the guidelines acknowledged that “evolution of AF-related thromboembolic risk evaluation is needed”.^50^ Some biomarkers may predict stroke or thromboembolic events in AF patients. Indeed, it has been shown that d-dimer,^16^^,^^51^ IL-6,^17^ CRP,^17^ NLR,^18^ NT-proBNP,^12^^,^^19^^,^^52^ troponin I,^19^ troponin T^13^ and vWf^14^ can act as additive prognostic markers to the CHA_2_DS_2_-VASc score for predicting stroke in AF subjects. Other studies have proposed new risk scores, such as the ABC score, which combines biomarkers with clinical factors and shows better predictive ability than the CHA_2_DS_2_-VASc score.^9^^,^^20^^,^^53^

Many recent studies have also shown the CHA_2_DS_2_-VASc score was associated with poor clinical outcomes such as mortality, and that the biomarkers mentioned above could provide additional accuracy for the prediction of mortality.^12^, ^13^, ^14^^,^^16^^,^^54^

The present findings provide further evidence that the CHA_2_DS_2_-VASc score is still an appropriate risk stratification scheme for stroke. CHA_2_DS_2_-VASc+SIS, which includes LMR, ALB and fibrinogen, may be a more practical risk stratification score for the prediction of mortality than the ABC score. Furthermore, several of the earlier studies were anticoagulation trials with carefully selected participants who all received oral anticoagulants and had more exclusion criteria than the present study.^13^^,^^16^^,^^17^^,^^19^^,^^52^ The participants in the hospital-based study had AF in real-life clinical practice. They tended to be older with more associated comorbidities, and with less use of oral anticoagulants. Therefore, the present study of 'real-world' AF patients adds some novel insights to current knowledge in this field, even though the sample size of this study was relatively small.

Some of the results on the c-statistic and NRI/IDI in the current study were inconsistent. The use of NRI and IDI methods to evaluate reclassification was recently criticized because of the risk of false-positive results.^55^^,^^56^ When the baseline model has a high c-statistic value, the c-statistic has low sensitivity for improving discrimination between the two models.^57^ Therefore, the improvement of the c-statistic was more conservative than NRI or IDI, and the authors considered it to be a more reliable index to evaluate the predictive ability of a new model.

### Limitations

Several limitations of this study should be considered when interpreting the results. Firstly, all cases and controls were recruited from a single tertiary medical center, and consequently, the findings may not be generalizable to other populations. Secondly, the sample size was relatively small and the follow-up time was not particularly long. However, this limitation would underestimate the association between the biomarkers and AF prognosis, suggesting the true association may be even greater than the present findings. Finally, the medications taken by the participants were likely to have changed during follow-up and may not have been captured by this study. Additional confounding factors are likely to be the increased use of warfarin or other oral anticoagulants during AF progression, leading to weaker associations between biomarkers and AF prognosis.

## Conclusions

This study found that LMR was the most informative biomarker for predicting stroke, while the strongest predictors of all-cause mortality were ALB, fibrinogen and LMR. The addition of LMR to the CHA_2_DS_2_-VASc score did not improve its predictive ability for stroke. However, the addition of SIS to the CHA_2_DS_2_-VASc score significantly improved its predictive value for all-cause mortality. These routinely available biomarkers may help to more accurately tailor the treatment options for individual patients with AF.

## Authors’ contributions

Conceptualization, Li Zhong, Yafei Li and Na Wu; Data curation, Long Wu, Zhiquan Yuan, Qin Hu, Huan Zhang, Chengying Li, Yanxiu Chen and Zhihui Zhang; Formal analysis, Huan Zhang and Na Wu; Supervision, Li Zhong, Yafei Li and Na Wu; Validation, Zhiquan Yuan and Huan Zhang; Writing-original draft, Long Wu, Yuhong Zeng, Lanqing Yang and Chengying Li; Writing-review & editing, Li Zhong, Yafei Li and Na Wu.

## Patient consent

Informed consent was obtained from all subjects involved in the study.

## Funding

This work was supported by the National Natural Science Foundation of China (NO.82073649 to N. W.) and the Natural Science Foundation Project of Chongqing, China (cstc2020jcyj-msxmX0105 to H.Q.).

## Ethics approval

The study was conducted according to the guidelines of the Declaration of Helsinki, and approved by the Ethics Committee of Southwest Hospital of Army Medical University (KY2020231).

## Data availability

The deidentified participant data will be shared on a request basis. Please directly contact the corresponding author to request data sharing.

## Declaration of competing interest

The authors declare no conflicts of interest.

## Appendix Supplementary materials

Image, application 1
