# Supplementary material for: Routinely available inflammation biomarkers to predict stroke and mortality in atrial fibrillation
Source: Clinics (Sao Paulo). 2025 Mar 11;80:100610. doi: 10.1016/j.clinsp.2025.100610 (PMC11950969; doi:10.1016/j.clinsp.2025.100610)
Supplement: Supplementary file 1 [file mmc1.pdf]

CLINICS-D-24-00689_Supplementary Material

Supplemental Table S1 The detailed cut-off values for biomarkers.

Biomarkers	The cut-off values for combined outcome events
CRP	≤ 12.0 mg/L
	> 12.0 mg/L
ALB	≤ 38.2 g/L
	> 38.2 g/L
Fibrinogen	≤ 3.3 g/L
	> 3.3 g/L
D-dimer	≤ 0.6 mg/L
	> 0.6 mg/L
PLR	≤ 91.2
	> 91.2
LMR	≤ 2.7
	> 2.7
NLR	≤ 4.1
	> 4.1

CRP, C-Reactive Protein; ALB, Albumin; PLR, Platelet to Lymphocyte Ratio; LMR, Lymphocyte to Monocyte Ratio; NLR, Neutrophil to Lymphocyte Ratio.

Supplemental Table S2 The detailed weighted points for the biomarkers in the final model for mortality.

**Biomarkers**	**Beta coefficient (β)**	**The quotient of β and the β whose absolute value is the smallest**	**Rounded the quotient up to the nearest integer**	**Weighted point (λ)**
All-cause mortality				
ALB	-1.143	-1.061	-1	-1
Fibrinogen	1.077	1	1	1
LMR	-1.470	-1.365	-1	-1

ALB, Albumin; LMR, Lymphocyte to Monocyte Ratio.

Supplemental Table S3 The detailed method to construct systemic inflammation score for all-cause mortality.

**Biomarkers**	**The scores for each biomarker**
ALB	
≤ 38.2 g/L	1
> 38.2 g/L	0
Fibrinogen	
≤ 3.3 g/L	0
> 3.3 g/L	1
LMR	
≤ 2.7	1
> 2.7	0

According to Table S2, the weighted points of three biomarkers for all-cause mortality are the same. ALB and LMR were negatively associated with the risk of all-cause mortality. So levels under the cutoff values were assigned as 1 for ALB and LMR, while as 0 for fibrinogen.

Supplemental Table S4 Association of inflammation biomarkers with adverse events in sensitivity analysis.

	Model 1[a]			Model 2[b]		
	**Beta coefficients**	**Adjusted HR (95% CI)**	**p-value**	**Beta coefficients**	**Adjusted HR (95% CI)**	**p-value**
**All-cause mortality**						
ALB						
≤ 38.2g/L		Reference			Reference	
> 38.2g/L	-1.14	0.32 (0.11, 0.95)	**0.041**	-1.14	0.32 (0.11, 0.95)	**0.041**
Fibrinogen						
≤ 3.3g/L		Reference			Reference	
> 3.3g/L	1.08	2.94 (1.23, 7.03)	**0.016**	1.08	2.94 (1.23, 7.03)	**0.016**
LMR						
≤ 2.70		Reference			Reference	
> 2.70	-1.47	0.23 (0.09, 0.62)	**0.003**	-1.47	0.23 (0.09, 0.62)	**0.003**
**Stroke**						
LMR						
≤ 2.70		Reference			Reference	
> 2.70	-1.683	0.19 (0.07, 0.47)	**<0.001**	-1.215	0.30 (0.10, 0.87)	**0.026**

HR, Hazard Ratio; ALB, Albumin; LMR, Lymphocyte to Monocyte Ratio.

The p-value with bold font indicate p-value < 0.05.

[a] Model 1 is Cox proportional hazards model for stroke adjusted for age, drink status, history of diabetes, history of previous stroke, right atrium diameter, left atrium diameter and these 7 biomarkers using a backward selection strategy. For all-cause mortality, adjusted for age, history of heart failure, left atrium diameter, left ventricular diameter, right atrium diameter, right ventricular diameter and these 7 biomarkers using a backward selection strategy.

[b] Model 2 is Cox proportional hazards model for stroke adjusted for age, drink status, history

of diabetes, history of previous stroke, right atrium diameter, left atrium diameter and these 7 biomarkers using a forward selection strategy. For all-cause mortality, adjusted for age, history of heart failure, left atrium diameter, left ventricular diameter, right atrium diameter, right ventricular diameter and these 7 biomarkers using a forward selection strategy.
